# Health-related quality of life among patients with treated alcohol use disorders, schizophreniform disorders or affective disorders and the influence of flexible and integrative psychiatric care models in Germany (PsychCare)

**DOI:** 10.3389/fpsyt.2023.1068087

**Published:** 2023-03-31

**Authors:** Anne Neumann, Bettina Soltmann, Roman Kliemt, Ines Weinhold, Jochen Schmitt, Andrea Pfennig, Fabian Baum

**Affiliations:** ^1^Center of Evidence-Based Health Care, Medizinische Fakultät Carl Gustav Carus, Technische Universität Dresden, Dresden, Germany; ^2^Department of Psychiatry and Psychotherapy, Carl Gustav Carus University Hospital, Technische Universität Dresden, Dresden, Germany; ^3^WIG2 Scientific Institute for Health Economics and Health System Research, Leipzig, Germany

**Keywords:** health-related quality of life, mental health care, psychiatric care models, affective disorders, schizophreniform disorders, alcohol use disorders, flexible and integrative care, symptom severity

## Abstract

**Introduction:**

Flexible and integrated treatment options (FIT) have been established in German psychiatric hospitals to enhance continuous and patient-centered treatment for patients with mental disorders. We hypothesized that patients with experience in FIT treatment showed higher health-related quality of life (HRQoL) and comparable symptom severity compared with patients treated as usual (TAU). Further, we expected that some sub-dimensions of HRQoL determined HRQoL results clearer than others, while certain factors influenced HRQoL and symptom severity stronger in the FIT compared to the TAU group. In addition, we hypothesized that HRQoL is correlated with symptom severity.

**Methods:**

We undertook a controlled, prospective, multicenter cohort study (PsychCare) conducted in 18 psychiatric hospitals in Germany, using the questionnaires Quality of Well Being Self-Administered (QWB-SA) (HRQoL) and Symptom-Checklist-K-9 (SCL-K-9) (symptom severity) at recruitment (measurement I) and 15 months later (measurement II). We assessed overall HRQoL (measured in health utility weights (HUW) and symptom severity score for patients from FIT and TAU treatment. We investigated the QWB-SA dimensions and separated the results by diagnosis. We used beta regressions to estimate the effect of multiple co-variates on both outcomes. To investigate the correlation between HRQoL and symptom severity, we used Pearson correlation.

**Results:**

During measurement I, 1,150 patients were recruited; while 359 patients participated during measurement II. FIT patients reported higher HUWs at measurement I compared to TAU patients (0.530 vs. 0.481, *p* = 0.003) and comparable HUWs at measurement II (0.581 vs. 0.586, *p* = 0.584). Symptom severity was comparable between both groups (I: 21.4 vs. 21.1, *p* = 0.936; II: 18.8 vs. 19.8, *p* = 0.122). We found lowest HRQoL and highest symptom severity in participants with affective disorders. HRQoL increased and symptom severity decreased over time in both groups. The QWB-SA dimension *acute and chronic symptoms* was associated with highest detriments in HRQoL. We identified risk/protective factors that were associated with lower quality of life and higher symptom severity in both groups. We confirmed that HRQoL was negatively associated with symptom severity.

**Discussion:**

Health-related quality of life (during hospital treatment) was higher among patients treated in FIT hospitals compared to patients in routine care, while symptom severity was comparable between both groups.

## 1. Introduction

Mental disorders are associated with decreased health-related quality of life (HRQoL) (1, 2). HRQoL is a comprehensive generic construct and covers many dimensions such as psychological status, functional abilities, subjective wellbeing, social interactions, role performance and physical health (3, 4). Generic instruments that measure HRQoL allow comparing the results across somatic and psychiatric diseases strengthening evidence-based decision-making in health policy and medical practice across diseases and disciplines. HRQoL is reduced among patients with an alcohol use disorder, schizophreniform disorders or affective disorders in comparison to the general population (2, 5–10). In addition, higher symptom severity has been found to be negatively related to HRQoL (11–13). Quality of life is an important concept in mental health care. There is an increasing focus on improving HRQoL, especially in patients with long term impairment and chronic illness (14).

### 1.1. Flexible and integrated treatment programs (FIT)

Mental health care services in German standard care are very heterogeneous as different cost units and service providers are involved based on different laws with a strong separation between outpatient and inpatient treatment and remuneration (15). The financial sector separation can lead to incentives for hospitals to maximize reimbursement, which then results in less than optional care for patients. The sectoral boundaries are particularly noticeable in the case of mental disorders (16). At the same time, patients with mental disorders in particular need continuous and patient-centered treatment. Therefore, innovative flexible and integrated treatment programs (FIT) for mental health care have been established and tested in several German psychiatric hospitals since 2013. Such FIT programs were established *via* specific contracts between psychiatric hospitals and health insurance providers. The remuneration of such contracts is based on Global Treatment Budgets (GBT). A GBT is a prospectively fixed budget covering a patient’s treatment in the psychiatric hospital independent from the type or setting of treatment {[inpatient, day care or outpatient as psychiatric outpatient department “Psychiatrische Institutsambulanz” (PIA)]} (17). FIT hospitals receive a fixed remuneration independent from the setting, duration or type of treatment, as long as the number of patients treated is in a specified range. Such remuneration enhances flexible and cross-sectoral care and shifts patients from inpatient to daycare or outpatient treatment. Common FIT concepts are, e.g., case managers, crisis resolution teams, assertive community treatment or treatment groups based on diagnoses not on setting. Patients who are not treated in FIT hospitals receive routine care, i.e., treatment as usual (TAU), with remuneration based on the costs and treatment per setting. As at 2021, 22 of such FIT hospitals have been introduced at German psychiatric hospitals. Previous research has shown that the introduction of FIT programs led to a reduced number of inpatients days and a shift to either increased day care and/or outpatient treatment in the hospital (18–20). However, the influence of such FIT programs on HRQoL is still unclear.

### 1.2. Hypotheses

We hypothesized that patients with experience in FIT treatment showed higher HRQoL and comparable symptom severity compared with patients treated as usual. Further, we expected that some sub-dimensions of HRQoL determined HRQoL results clearer than others, while certain factors influenced HRQoL and symptom severity stronger in the FIT compared to the TAU group. In addition, we hypothesized that HRQoL is correlated with symptom severity. HRQoL was in the focus of this manuscript, while symptom severity was mainly used to describe the study population and the interrelationship with HRQoL.

## 2. Materials and methods

### 2.1. Study design

We present results from the PsychCare study, which is a controlled, prospective, multicenter cohort study conducted in 18 psychiatric hospitals in Germany. For more details on the study design, we published the study protocol elsewhere (21). For the identification of participating FIT hospitals, we ranked all FIT hospitals as of 2017 in a randomized manner stratified by the year of FIT onset (before vs. after 2015). Based on this ranked list, we consecutively asked hospitals for their participation in our study. For each participating FIT hospital, we identified structurally comparable psychiatric hospitals for the TAU group (treatment as usual, more details on hospital selection below in “routine care”) and consecutively asked for their participation in our study.

Participating hospitals recruited all patients who fulfilled all inclusion and none of the exclusion criteria (see below in “inclusion and exclusion criteria”) from February 2018 until September 2019 (measurement I). Consequently, we allocated the participants in either FIT or TAU group on the hospital level. Therefore, if a patient was treated in a FIT or TAU hospital within the recruitment period, the patient was asked to participate in our study and remained in this group (FIT vs. TAU), independent from the length of treatment within the group. We collected data on the same outcomes, including HRQoL and symptom severity, at two measurement time points. Measurement I took place in participating hospitals at recruitment and measurement II 15 months later through written contact (with patients at home). We included all patients from measurement I, independent from whether they participated in the measurement II, and analyzed both time points separately. Our *a priori* sample size calculation for the QWB-SA (primary outcome) estimated 153 participants for each treatment and diagnosis group (estimated effect size: 0.33, difference: 0.05, SD: 0.16; estimated lost-to-follow-up: 25%; α 5%; power: 80%).

### 2.2. Inclusion and exclusion criteria

We included patients if they,

– had received in- or outpatient treatment in one of the participating hospitals during recruitment phase,
– were at least 18 years of age,
– had any of the following clinical diagnoses according to the International Classification of Disease, 10th version (ICD-10) (22): mental and behavioral disorders due to use of alcohol (F10, abbreviated to “alcohol use disorders”), schizophrenia, schizotypal disorder, delusional disorders or brief psychotic disorders (F20-23, abbreviated to “schizophreniform disorders”), or affective disorders (F30-39),
– showed sufficient command of German language to take part in the study and,
– provided informed consent.

We excluded patients from the study if they,

– had severe organic brain dysfunction including impairment of cognitive function,
– had severe intellectual disabilities or,
– showed acute suicidality.

The entire PsychCare study population also included younger patients (6–17 years) and other diagnoses (ICD-10: F50, F90-98). However, due to the low number of recruited patients with younger age (*n* = 58) and other diagnoses (*n* = 21), we excluded them from the following analyses.

### 2.3. Routine care

We defined eligible hospitals for the TAU group *a priori* by selecting those being in the same region, having a psychiatric in- and outpatient unit and not having a FIT-like contract. We matched the structural comparability of the hospitals based on data from structured quality hospital reports and the German spatial sociodemographic and socioeconomic database INKAR (Indicators and Maps for Spatial and Urban Development) (23). We based hospital allocation on the number of cases per diagnosis with a weighting of 50%, structural features of hospitals (e.g., number of beds or number of personnel) with a weighting of 25%, and regional factors (e.g., unemployment rate or household income) with a weighting of 25%. For more details on the selection of control hospitals, see the methods description in Petzold et al. (24) and the PsychCare study protocol (21).

### 2.4. Outcome measures

We used the results from the German versions of the questionnaires Quality of Well Being Self-Administered (QWB-SA) (25) to examine HRQoL and the Symptom-Checklist-K-9 (SCL-K-9) (26) to examine symptom severity at measurement I and measurement II, and compared them for both groups at both time points.

#### 2.4.1. HRQoL

Health-related quality of life is one of two primary outcome measures in the PsychCare study. We used the QWB-SA questionnaire to measure HRQoL as used in other studies (27, 28). The QWB-SA is a preference-based and self-administered instrument to describe HRQoL measured as health utility weights (HUW). The QWB-SA was selected for this study, as it is a short, generic and preference-based instrument to assess the HRQoL. It, therefore, does not overload the study participants together with the other instruments used and can measure the patients’ health utility for health economic evaluations examined in another part of the PsychCare study. The QWB-SA combines three scales of functioning with a measure of symptoms and problems to estimate a point-in-time expression of wellbeing that runs from 0 (equivalent to death) to 1 (equivalent to perfect health) (29). The instrument includes four dimensions. The first dimension describes *acute and chronic symptoms (CPX dimension)*, i.e., the presence or absence of 19 chronic symptoms or problem, followed by 25 acute (or more transient) physical symptoms and 14 mental health symptoms and behaviors (29). The second dimension describes *self-care* aspects and a person’s *mobility (MOB dimension).* The third dimension assessed *physical activity (PAC dimension)*. And the fourth dimension describes *self-care aspects* and *usual activity* (SAC dimension) including completion of role expectations (29). We calculated the HUW of the QWB-SA by subtracting the maximum weighted item of each dimension for each of the last 3 days from the perfect score 1.0. We then added the daily QWB scores and divided this sum by three to obtain the average self-administered QWB score, the HUW. More information on the scoring can be obtained from the Coding and Scoring Manual (30).

#### 2.4.2. Symptom severity

In addition, we assessed symptom severity using the SCL-K-9 questionnaire. The SCL-K-9 is a short form of the SCL-90-R to define the subjective burden of psychological symptoms. The SCL-90-R was first described by Derogatis (31) and includes 90 items which define the global extent of psychological symptoms on self-rater basis. However, it has been found that 90 items might be too lengthy and time-consuming, especially for (severely) mentally ill patients (26, 32, 33). Therefore, we chose the SCL-K-9 instrument. The SCL-K-9 has been proven to reliably measure the global extent of symptom severity (34, 35). It describes the amount of psychological complaints as well as global distress (36). The SCL-K-9 uses nine items, one item from each of the nine scales of the SCL-90-R, which showed the greatest discriminant power to the average psychological distress level (GSI-90) in a representative German survey (33). The nine items are: uncontrollable emotional outbursts, finding it difficult to start something, feeling that you worry too much, emotional vulnerability, feeling observed or talked about, feeling uptight or agitated, feeling of heaviness in your arms and legs, feeling nervous when left to yourself, feelings of loneliness even in company. The instrument rates each item using a 5-point Likert scale from “not at all” to “very much.” We charged all answers with 1 to 5 each and summed them up to a SCL-K-9 sum score (theoretical range: 9–45). A high sum score indicates high and a low sum score low symptom severity. More information on the instrument and scoring can be obtained elsewhere (26, 37).

### 2.5. Co-variates

We used the following co-variates in our analyses:

• age (additionally grouped in to the following three categories: 18–39, 40–59, ≥60 years),
• sex (female, male),
• diagnosis at study entry,
• partnership status (married or co-habiting vs. not),
• accommodation (supported living vs. not),
• living situation (living alone vs. not),
• education (lower, intermediate or higher; definition see below),
• occupation (working, not working, incapacitated or unable to work; definition see below),
• time in treatment (at least 5 years of psychiatric treatment vs. less than 5 years) and,
• chronic disease (any chronic disease vs. no; definition see below).

All co-variates were reported at measurement I (recruitment).

We measured education according to the Comparative Analysis of Social Mobility in Industrial Nations (CASMIN) scale (38), translated and adapted to the German context (39). The CASMIN scale is a combination of the attained general education and vocational education based on the reached degree. We used the highest education and occupation reported by the study participants. We grouped the CASMIN into three categories in line with Leopold (40). The category “lower education” comprises study participants holding lower secondary degrees (9 years of schooling) with completed vocational qualification or less (CASMIN 1a–1c). “Intermediate education” ranges from intermediate secondary degrees (at least 10 years of schooling) to higher secondary degrees with vocational qualification (CASMIN 2a–2cvoc). The category “higher education” includes individuals holding tertiary degrees (CASMIN 3a–3b). We omitted participants with missing data from this categorization and separately stated those who were in education (39).

We classified occupation into working (full-time, part-time, other employment), not working (unemployed, pension, housewife/househusband and undergoing training) and incapacitated or unable to work similar to Huber et al. (41). If the study participants indicated more than one mentioned category, we included them in the highest category according to the sequence of the previous sentence.

For the definition of chronic disease, we exclusively considered somatic chronic diseases to avoid correlation with the diagnoses under investigation. Chronic diseases were defined as hypertension (ICD-10: I10-I15), diabetes (E10-E14), heart disease (I05-I09, I30-I52), gastrointestinal diseases (K50, K51), migraine (G43, G44), cancer (C00-C97), thyroid disease (E00-E07), and musculoskeletal disorders (M00-M19, M79.0, M79.1, M80-M82) similar to Domenech et al. (12) and Huber et al. (41).

### 2.6. Analyses

We assessed overall HUW and symptom severity score at measurement I and measurement II for patients from FIT hospitals contrasting patients from TAU, and by diagnosis at admission. In addition, we visualized the results by diagnosis using box plots to present median, upper and lower quartiles as well as outliers.

The time span between the date when measurement I questionnaire was completed and the date when measurement II questionnaire was completed, varied between study participants (400–825 days, mean = 497 days). If participants completed the measurement II questionnaire later than 850 days after measurement I, we did not consider this questionnaire at all (*n* = 1). We assessed each time point separately, in contrast to a longitudinal design, as measurement I was conducted already under interventional circumstances, i.e., FIT implementation for at least 2 years at measurement I. Therefore, we expected intervention effects already at measurement I. Results of the follow-up examination are additionally important as the treatment circumstances and disease severity differed between measurement I and measurement II. Recruitment at measurement I took place at either inpatient, day care or outpatient hospital treatment, while measurement II was independent from treatment in a hospital setting.

In order to assess which aspects were associated with the highest detriments in HRQoL and whether there are differences between the included diagnostic groups, we further investigated the role of the four dimensions of the QWB-SA questionnaire and separated the results by diagnosis. We used beta regressions (42) to estimate the effect of multiple co-variates on HUW, as the distribution of the HUW fitted well with the beta distribution. Beta regression has been applied in several studies on HRQoL (43–47). As covariates were present, we used the alternative parameterization with location parameter and scale parameter (47). The mean HUW is represented by μ (alternative parameterization). We did not adjust our regression analysis to the variable “setting” (inpatient, daycare and outpatient) as this variable is only valid during measurement I. Moreover, this variable is a self-explanatory feature (model effect) and, therefore, could distort the regression results due to its correlation with the group (FIT vs. TAU) variable. Therefore, we added a sub-analysis by setting for HUW at measurement I independent from the group (see Supplementary material). Further, as the number of patients, who did not participate in measurement II, was quite high, we additionally reported the HUWs of those who participated in both measurements as part of the sensitivity analyses (see Supplementary material). As the distribution of the symptom severity score fitted well with the normal distribution, we used linear regression to estimate the effect of multiple co-variates on symptom severity. We conducted each regression by each diagnosis group separately and adjusted by each co-variate (see Table 1), excluding diagnosis and setting. To investigate the correlation between HRQoL and symptom severity we used the Pearson correlation coefficient. We applied 5% as the level of significance and conducted all statistical analyses using the statistical software R V.4.0.3 (48).

**TABLE 1:** Study population, all characteristics at the time of study inclusion.

	Patients, who completed measurement I		Patients, who completed measurement I and II	
	**FIT (*N* = 595)**	**TAU (*N* = 555)**	**FIT (*N* = 217)**	**TAU (*N* = 142)**
**Age**				
Mean (SD)	45.7 (14.2)	47.5 (15.2)	47.3 (13.3)	47.6 (13.9)
Age group				
18–39	208 (35.0%)	182 (32.8%)	58 (26.7%)	42 (29.6%)
40–59	303 (50.9%)	269 (48.5%)	128 (59.0%)	80 (56.3%)
≥60	82 (13.8%)	104 (18.7%)	31 (14.3%)	20 (14.1%)
Missing	2 (0.3%)	–	–	–
**Sex**				
Female	312 (52.4%)	271 (48.8%)	123 (56.7%)	79 (55.6%)
Missing	1 (0.2%)	1 (0.2%)	–	–
**Diagnosis**				
Alcohol use disorders	125 (21.0%)	138 (24.9%)	44 (20.3%)	30 (21.1%)
Schizophreniform disorders	117 (19.7%)	85 (15.3%)	46 (21.2%)	20 (14.1%)
Affective disorders	353 (59.3%)	332 (59.8%)	127 (58.5%)	92 (64.8%)
**Partnership status**				
Not married/co-habiting	442 (74.3%)	404 (72.8%)	155 (71.4%)	97 (68.3%)
Missing	11 (1.8%)	19 (3.4%)	–	–
**Accommodation**				
Supported accommodation	22 (3.7%)	21 (3.8%)	7 (3.2%)	4 (2.8%)
Missing	11 (1.8%)	15 (2.7%)	1 (0.5%)	5 (3.5%)
**Living situation**				
Living alone	257 (43.2%)	242 (43.6%)	100 (46.1%)	55 (38.7%)
Missing	57 (9.6%)	52 (9.4%)	15 (6.9%)	12 (8.5%)
**Education**				
Higher: tertiary degree	91 (15.3%)	88 (15.9%)	37 (17.1%)	32 (22.5%)
Intermediate: secondary degree	313 (52.6%)	275 (49.5%)	117 (53.9%)	77 (54.2%)
Lower: lower secondary degree	139 (23.4%)	137 (24.7%)	48 (22.1%)	22 (15.5%)
Currently in training	39 (6.6%)	37 (6.7%)	14 (6.5%)	5 (3.5%)
Missing	13 (2.2%)	18 (3.2%)	1 (0.5%)	6 (4.2%)
**Occupation**				
Working	254 (42.7%)	203 (36.6%)	99 (45.6%)	60 (42.3%)
Not working	213 (35.8%)	235 (42.3%)	66 (30.4%)	58 (40.8%)
Incapacitated or unable to work	86 (14.5%)	76 (13.7%)	44 (20.3%)	15 (10.6%)
Missing	42 (7.1%)	41 (7.4%)	8 (3.7%)	9 (6.3%)
**Time in treatment**				
>5 years	388 (65.2%)	359 (64.7%)	147 (67.7%)	105 (73.9%)
**Chronic comorbidity**				
At least one chronic disease	207 (34.8%)	196 (35.3%)	38 (17.5%)	53 (37.3%)
**Setting**				
Inpatient	170 (28.6%)	404 (72.8%)		
Day care	292 (49.1%)	121 (21.8%)		
Outpatient	29 (21.7%)	12 (2.2%)		
Missing	4 (0.7%)	18 (3.2%)		

FIT, participants from FIT hospitals; TAU, participants from routine care; measurement II = assessment at 15 months after measurement I; diagnosis at study entry (ICD-10): alcohol use disorders (ICD-10: F10) = mental and behavioral disorder due to use of alcohol, schizophreniform disorders (ICD-10: F20-23) = schizophrenia, schizotypal disorder, delusional disorder or brief psychotic disorder.

## 3. Results

### 3.1. Characteristics of study participants

Of the 1,150 (FIT: 595; TAU: 555) patients who participated in the PsychCare study during measurement I, 1,084 (FIT: 568; TAU: 516) patients completed the QWB-SA and 1,099 (FIT: 576; TAU: 523) patients the SCL-K-9. Of the 359 (FIT: 217; TAU: 142) patients who participated during measurement II, 339 (FIT: 205; TAU: 134) patients completed the QWB-SA and 348 (FIT: 209; TAU: 139) patients the SCL-K-9. Only 36.5% (FIT) and 25.6% (TAU) of those participating in measurement I also took part in measurement II.

Participants, who completed measurement I, had a mean age of 45.7 (FIT) and 47.5 (TAU) years (Table 1). More than half had an affective disorder diagnosis (FIT: 58.1%; TAU: 59.0%) and had been ill for more than 5 years (FIT: 65.2%; TAU: 64.7%) (Table 1). Participants, who completed measurement I and II, had a mean age of 47.3 (FIT) and 47.6 (TAU), were predominantly diagnosed with affective disorders (FIT: 58.5%; TAU: 64.8%) and had mainly been treated more than 5 years (FIT: 67.7%; TAU: 73.9%).

In sum, the FIT and TAU populations are comparable for further analyses.

### 3.2. HRQoL and symptom severity

**FIGURE 1:**
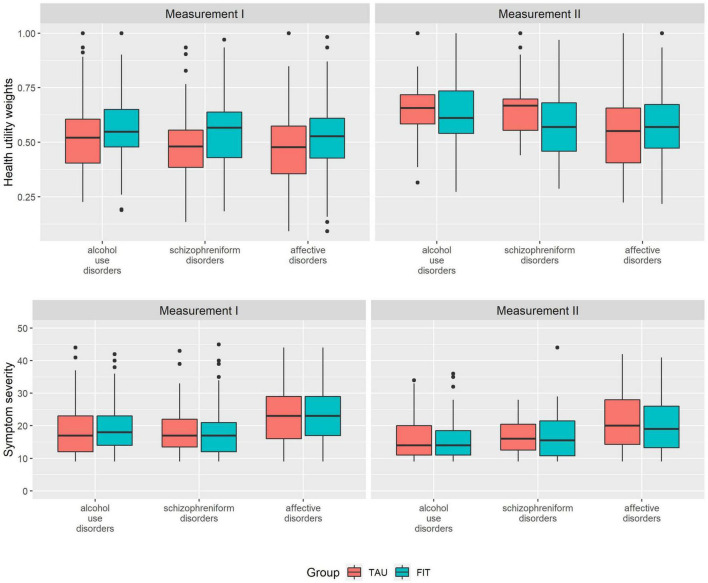
Health utility weights and symptom severity scores at measurement I and measurement II, by diagnosis at study entry. FIT = participants from FIT hospitals (flexible and integrated treatment), TAU = participants from routine care; measurement II = examination at 15 months after measurement I; diagnosis at study entry (ICD-10): Alcohol use disorders (ICD-10: F10) = mental and behavioral disorder due to use of alcohol, schizophreniform disorders (ICD-10: F20-23) = schizophrenia, schizotypal disorder, delusional disorder or brief psychotic disorder.

The HUW during measurement I was higher among patients experiencing FIT treatment compared to TAU (0.530 vs. 0.481) (Table 2). This difference was statistically significant (see Table 3, group). A higher HUW among participants from FIT-hospitals could also be found in all diagnosis groups (Figure 1). Participants with F10 had the lowest HUW compared with the other diagnostic groups. During measurement II, however, participants with alcohol use disorders or schizophreniform disorders showed even lower HUW compared to participants from the TAU group. These differences were, however, not statistically significant (Table 3, see variable “group”). In addition, HUW at measurement I were lowest among patients recruited in an inpatient setting, followed by day care and highest for those recruited in an outpatient setting (see Supplementary Table A). Our sensitivity analysis revealed that among those who participated in both measurements, HUW was slightly higher during measurement I in both groups (Supplementary Table B) compared to all who participated in measurement I (Table 2). However, the HUW was again significantly higher in the FIT compared to the TAU group (Supplementary Table B).

**TABLE 2:** Health utility weights and symptom severity scores at measurement I and measurement II, by diagnosis at study entry.

	Measurement I		Measurement II	
	**FIT**	**TAU**	**FIT**	**TAU**
**Mean health utility weights (SD)**	*n* = 568	*n* = 516	*n* = 205	*n* = 134
Overall	0.530 (0.149)	0.481 (0.154)	0.581 (0.156)	0.586 (0.172)
**By diagnosis at study entry**				
Alcohol use disorders	0.562 (0.149)	0.517 (0.151)	0.639 (0.165)	0.651 (0.146)
Schizophreniform disorders	0.547 (0.156)	0.488 (0.162)	0.570 (0.150)	0.663 (0.156)
Affective disorders	0.513 (0.144)	0.465 (0.151)	0.565 (0.152)	0.550 (0.173)
**Mean symptom severity (SD)**	*n* = 576	*n* = 523	*n* = 209	*n* = 139
Overall	21.4 (8.08)	21.1 (8.07)	18.8 (7.86)	19.8 (8.47)
**By diagnosis at study entry**				
Alcohol use disorders	19.2 (7.40)	18.4 (7.48)	16.3 (7.10)	16.9 (7.96)
Schizophreniform disorders	18.2 (7.60)	18.5 (7.28)	17.2 (7.35)	16.7 (5.58)
Affective disorders	23.3 (7.94)	23.0 (8.02)	20.3 (8.00)	21.4 (8.79)

FIT = participants from FIT hospitals (flexible and integrated treatment), TAU = participants from routine care; measurement II = examination at 15 months after measurement I; diagnosis at study entry (ICD-10): alcohol use disorders (ICD-10: F10) = mental and behavioral disorder due to use of alcohol, schizophreniform disorders (ICD-10: F20-23) = schizophrenia, schizotypal disorder, delusional disorder or brief psychotic disorder.

**TABLE 3:** Regression analyses, health utility weights and symptom severity, at measurement I and measurement II, by diagnosis at study entry.

Main analysis								
	**Health utility weights**				**Symptom severity**			
	**Measurement I**		**Measurement II**		**Measurement I**		**Measurement II**	
	**Estimate**	***P*-value**	**Estimate**	***P*-value**	**Estimate**	***P*-value**	**Estimate**	***P*-value**
Intercept	0.61	<**0**.**001**	0.74	<**0**.**001**	19.35	<**0**.**001**	18.4	<**0**.**001**
**Group (Reference: TAU)**								
FIT	0.15	**0**.**003**	0.06	0.584	0.04	0.936	-1.54	0.122
**Age group (Reference: 18–39 years)**								
40–59 years	0.08	0.176	-0.13	0.326	-2.04	**0**.**001**	-1.47	0.212
≥ 60 years	-0.05	0.532	-0.06	0.733	-3.937	<**0**.**001**	-2.27	0.159
**Sex (Reference: male)**								
Women	-0.11	**0**.**026**	-0.5	<**0**.**001**	1.88	<**0**.**001**	2.55	**0**.**007**
**Partnership status (Reference: married, co-habiting)**								
Not married/co-habiting	-0.23	**0**.**001**	0.5	**0**.**002**	0.21	0.775	-0.76	0.581
**Accommodation (Reference: independent)**								
Supported accommodation	0.14	0.631	-0.24	0.798	-2.3	0.437	5.53	0.497
**Living situation (Reference: not living alone)**								
Living alone	0.04	0.486	-0.45	**0**.**003**	-0.44	0.493	1.05	0.407
**Education (Reference: higher: Tertiary degree)**								
Intermediate: Secondary degree	-0.24	<**0**.**001**	-0.48	<**0**.**001**	1.26	0.076	2.53	**0**.**033**
Lower: lower secondary degree	-0.15	0.051	-0.3	0.081	1.27	0.121	3.14	**0**.**031**
Currently in training	-0.22	**0**.**048**	-1.05	<**0**.**001**	2.16	0.066	4.25	0.076
**Occupation (Reference: working)**								
Not working	-0.07	0.188	0.03	0.794	-0.27	0.630	0.62	0.561
Incapacitated or unable to work	-0.13	0.097	-0.42	**0**.**008**	0.56	0.475	1.96	0.157
**Chronic disease (Reference: no chronic disease)**								
Chronic comorbidity	-0.28	<**0**.**001**	-0.43	<**0**.**001**	1.92	**0**.**001**	0.92	0.395
**Time in treatment (Reference: ≤ 5 years)**								
> 5 years	-0.18	<**0**.**001**	0.03	0.809	1.68	**0**.**002**	1.25	0.226
**Sub-analysis by diagnostic groups**								
**Alcohol use disorders**								
Intercepts	0.19	**0**.**021**	0.83	<**0**.**001**	18.42	<**0**.**001**	16.93	<**0**.**001**
Group (Reference: TAU)								
FIT	0.27	**0**.**02**	0.09	0.75	0.75	0.422	-0.63	0.727
**Schizophreniform disorders**								
Intercept	-0.03	0.611	1.33	<**0**.**001**	18.46	<**0**.**001**	16.70	<**0**.**001**
Group (Reference: TAU)								
FIT	0.23	**0**.**013**	-1.08	<**0**.**001**	-0.23	0.832	0.5	0.786
**Affective disorders**								
Intercept	-0.08	0.035	0.37	<**0**.**001**	22.96	<**0**.**001**	21.36	<**0**.**001**
Group (Reference: TAU)								
FIT	0.13	**0**.**013**	-0.01	0.929	0.31	0.624	-1.07	0.358

FIT = participants from FIT hospitals (flexible and integrated treatment), TAU = participants from routine care; measurement II = examination at 15 months after measurement I; diagnosis at study entry (ICD-10): alcohol use disorders (ICD-10: F10) = mental and behavioral disorder due to use of alcohol, schizophreniform disorders (ICD-10: F20-23) = schizophrenia, schizotypal disorder, delusional disorder or brief psychotic disorder. Bold values represent statistical significance (*p* < 0.05).

Symptom severity was comparable between FIT and TAU, both at measurement I and measurement II, and highest among participants with affective disorders (Figure 1 and Table 2). Our sensitivity analysis showed that among those who participated in both measurements, symptom severity was slightly lower among FIT (seen in all diagnostic groups) and higher among TAU patients during measurement I (triggered by participants with affective disorder) (Supplementary Table B) compared to all who participated in measurement I (Table 2). The differences between the FIT and TAU group were again not significant (Supplementary Table B).

### 3.3. HRQoL by dimensions

The dimension associated with highest detriments was *acute and chronic symptoms*, independent from the diagnosis at study entry (Table 4). The dimensions *mobility*, *physical activity* and *usual activity* were associated with comparable detriments during measurement I; while during measurement II *physical activity* detriments were strongest among the three dimensions. During measurement I, all impairments in the dimensions *acute and chronic symptoms* and *mobility* among patients treated in FIT hospitals were smaller compared to patients in the TAU group.

**TABLE 4:** QWB-SA dimensions at measurement I and measurement II, by diagnosis at study entry.

	Measurement I		Measurement II	
**QWB-SA dimensions**	**FIT**	**TAU**	**FIT**	**TAU**
**Acute and chronic symptoms**	0.373 (0.113)	0.381 (0.122)	0.354 (0.110)	0.347 (0.129)
**By diagnosis at study entry**				
Alcohol use disorders	0.345 (0.126)	0.351 (0.120)	0.318 (0.134)	0.296 (0.145)
Schizophreniform disorders	0.356 (0.121)	0.388 (0.120)	0.349 (0.106)	0.307 (0.134)
Affective disorders	0.389 (0.102)	0.391 (0.121)	0.367 (0.100)	0.371 (0.116)
**Mobility**	0.028 (0.038)	0.050 (0.041)	0.004 (0.015)	0.006 (0.018)
**By diagnosis at study entry**				
Alcohol use disorders	0.034 (0.040)	0.056 (0.041)	0.002 (0.008)	0.005 (0.018)
Schizophreniform disorders	0.027 (0.036)	0.050 (0.041)	0.008 (0.023)	0.003 (0.009)
Affective disorders	0.026 (0.037)	0.048 (0.041)	0.003 (0.013)	0.007 (0.020)
**Physical activity**	0.035 (0.047)	0.042 (0.052)	0.041 (0.049)	0.042 (0.053)
**By diagnosis at study entry**				
Alcohol use disorders	0.030 (0.044)	0.035 (0.049)	0.027 (0.035)	0.025 (0.035)
Schizophreniform disorders	0.041 (0.045)	0.040 (0.052)	0.048 (0.051)	0.028 (0.049)
Affective disorders	0.035 (0.048)	0.045 (0.054)	0.043 (0.052)	0.050 (0.057)
**Usual activity**	0.025 (0.025)	0.024 (0.026)	0.019 (0.026)	0.018 (0.024)
**By diagnosis at study entry**				
Alcohol use disorders	0.016 (0.023)	0.015 (0.025)	0.013 (0.025)	0.008 (0.017)
Schizophreniform disorders	0.018 (0.024)	0.020 (0.024)	0.022 (0.026)	0.004 (0.008)
Affective disorders	0.030 (0.025)	0.029 (0.026)	0.021 (0.026)	0.024 (0.026)

FIT = participants from FIT hospitals (flexible and integrated treatment), TAU = participants from routine care; measurement II = examination at 15 months after measurement I; diagnosis at study entry (ICD-10): alcohol use disorders (ICD-10: F10) = mental and behavioral disorder due to use of alcohol, schizophreniform disorders (ICD-10: F20-23) = schizophrenia, schizotypal disorder, delusional disorder or brief psychotic disorder.

Among *acute and chronic symptoms*, participants with affective disorders showed the highest detriments in both groups and at both time points, followed by patients with schizophreniform disorders and alcohol use disorders. Among the dimension *mobility*, we observed highest detriments among participants with alcohol use disorders during measurement II, while the impairments between the other two diagnoses during measurement I and among all diagnoses during measurement II were comparable. The absolute detriments during measurement II in this dimension were very small. Among *physical activity*, the highest impairments among patients in the FIT-group were for patients with schizophreniform disorders, while highest detriments among patients in the TAU-group can be observed among patients with affective disorders. Among the dimension *usual activity*, highest detriments were among patients with affective disorders, with the exception of the FIT-group during measurement II.

### 3.4. Influencing factors on and correlation between HRQoL and symptom severity

Our regression analyses (Table 3) show that the HUW during measurement I was significantly higher among patients in FIT hospitals compared to patients treated in the TAU-group. The HUW difference during measurement II and the differences regarding symptom severity were not significant (for descriptive results see Table 2).

Higher age was associated with lower symptom severity during measurement I. Women reported lower HUW and higher symptom severity at both time points compared to men. Not being married or co-habited compared to being married or co-habited decrease HUW during measurement I and increased it during measurement II. Living under supported accommodation situations did not significantly influence HUW nor symptom severity in our study. Those participants who lived alone during measurement II reported lower HUW compared to those who did not live alone. Not having higher education status resulted in lower HUW at both time points, whereas these results were only statistically significant for intermediate education and being currently in training compared to higher education. The comparison of lower education with higher education was, however, not significant. Not having higher education resulted in higher symptom severity compared to higher education during measurement II (currently in training was not significant). Being incapacitated or unable to work was an indicator for lower HUW during measurement II compared to those participants who were working. Having any chronic comorbidity decreased HUW at both time points and increased symptom severity during measurement I. Patients who were under psychiatric treatment for more than 5 years showed lower HUW and higher symptom severity during measurement I.

For each of the three diagnoses, HUW during measurement I was significantly lower among the FIT-group compared to TAU, while during measurement II this was only the case for patients with schizophreniform disorders. We could observe no significant difference between FIT and TAU regarding symptom severity considering each diagnosis group.

### 3.5. Correlation between HRQoL and symptom severity

Health utility weights und symptom severity were negatively correlated at measurement I and measurement II (Table 5), indicating a higher symptom load was associated with greater detriments in HRQoL. This effect was strong both at measurement I and measurement II according to Cohen’s statistic (49).

**TABLE 5:** Correlation between health utility weights and symptom severity, at measurement I and measurement II.

Measurement	*r*	df	*P*-value
Measurement I	−0.51	1,062	<0.001
Measurement II	−0.67	335	<0.001

r = correlation coefficient; df = degrees of freedom.

## 4. Discussion

### 4.1. Health related quality of life in general

The range of self-reported HUWs on measurement I and II suggests that the study participants have only about half the value for “self-perceived perfect health.” These values were consistent with and rather in the lower range of values among persons with psychiatric disorders as described in the literature (50–53). We expected that our study population would show slightly lower HRQoL compared to the general population with mental disorders as we recruited patients during treatment in a hospital setting. Based on clinical assessments, Kaplan et al. postulated a 0.03 change in score as a minimum clinically important difference (MCID), which was met here with a difference of 0.049 (54, 55).

We found the lowest HRQoL in participants with affective disorders followed by those with schizophreniform disorders and with alcohol use disorders, regardless of group membership. Other studies also reported diagnosis-specific differences of HRQoL (5, 56–60) and lowest HRQoL among patients with diagnoses in the area of affective disorders (5, 56, 57), whereas other studies observed more severe levels of disability among individuals with schizophrenia compared to those with bipolar affective disorders (59, 60). HRQoL increased over time in both groups. This is probably because recruitment was done during hospital stay or outpatient treatment at the hospital and, therefore, often during a rather acute phase of illness; while measurement II was 15 months after measurement I independent from the stage of illness. Consistent with this observation, symptom severity decreased over time. As hypothesized, HRQoL was negatively related to symptom severity as seen in other studies (11–13). Symptom severity was highest among participants with affective disorders compared to the other two diagnosis groups. As HRQoL and not symptom severity was in the focus of this manuscript, we mainly discuss the results of HRQoL.

### 4.2. Comparison of study population FIT and TAU

There were no significant differences between FIT and TAU in terms of sociodemographic and clinical characteristics such as age, gender, living conditions, diagnoses, and duration of illness. We only found two differences between the groups at measurement I. First, the proportion not working was slightly smaller among FIT patients, which could hint to a somewhat less severely ill FIT population. However, this was not supported by other sociodemographic factors. Second, fewer patients were recruited during inpatient and more during day care or outpatient treatment among FIT hospitals compared to the TAU group. This difference can be explained by the lower intensity of inpatient and higher intensity of day care and outpatient treatment in FIT hospitals, which can be considered as an interventional effect in itself, introduced by FIT programs, as seen in other studies (18, 19, 61–63). In addition, recruitment across all settings might be facilitated in FIT hospitals as settings are blurred in FIT hospitals due to re-structuring within FIT hospitals, while different staff is often responsible for different settings making recruitment across settings difficult in routine care.

### 4.3. Comparison of results between FIT and TAU

Even though patient characteristics and symptom severity were comparable between patients experiencing FIT treatment and TAU, HRQoL at measurement I was higher among FIT patients. We observed this difference in all diagnosis groups. Both observations were also valid considering only those patients that completed the questionnaire at both time points. Our data shows lowest HRQoL at measurement I during recruitment at inpatient stay followed by daycare treatment (see Supplementary material). As mentioned above, fewer patients were recruited in an inpatient setting in the FIT group compared to TAU, while the percentage recruited during day care or outpatient treatment was higher in the FIT group. Therefore, the difference in HUW at measurement I can also be associated with the different recruitment in the settings between FIT and TAU. On the other hand, the concept of inpatient, day care vs. outpatient is less structured in FIT hospitals and therefore a less clear classification in comparison to the TAU group.

One of the reasons why the differences in HRQoL between the groups decreased over time could be related to the COVID-19 pandemic. The majority of the measurement II was conducted in March 2020 or later, during the first and second waves of the corona pandemic in Germany (64). The associated restrictions and countermeasures affected all areas of health care. Of particular note, however, were the restrictions and closures in the area of day hospitals (65). Since FIT hospitals reduced the number of beds already before the corona pandemic and partly shifted to the day clinic area, which was structurally anchored, the closure of the day clinics probably had a greater impact on patient care in FIT hospitals than in TAU hospitals. On the other hand, whether FIT hospitals may have been able to act more flexibly in the COVID-19 pandemic because of their more flexible structuring is uncertain. Studies on the effects of the COVID-19 pandemic on the care in FIT vs. TAU hospitals are still pending. Further research is needed in this area.

Another reason might be a ceiling effect of HUW that might have been reached in both groups during measurement II and higher HRQoL cannot be expected in our study population.

### 4.4. Aspects influencing HRQoL

The dimension that was associated with highest detriments was *acute and chronic symptoms*, independent from the diagnosis at study entry. The other dimensions (*mobility*, *physical activity* and *usual activity*) only added lower detriments. We expected that the presence of the 19 chronic symptoms or problems (e.g., blindness and speech problems), together with the followed 25 acute (or more transient) physical symptoms (e.g., headache, coughing and pain) as well as the 14 mental health symptoms and behaviors (e.g., sadness, anxiety and irritation) would be associated with the highest detriments in HRQoL, especially in our study population (29). Symptoms are known to be correlated with HRQoL (66–68). In our study, symptom severity was also negatively correlated with HRQoL underlying the importance of this dimension in the QWB-SA instrument.

### 4.5. Influence of risk factors

In our study, higher age was associated with higher symptom severity during measurement I, while age did not significantly influence HRQoL. In line with our finding, other studies also reported a lack of or only a small association of age on HRQoL (5, 69–72). In contrast, in the German general population, HRQoL was reported to decrease with age (41). This age difference in the general population might diminish focusing on our selected diseases as the detriments of the mental disorders might overweight any age difference or make such differences smaller.

Women in our study consistently reported lower HRQoL and higher symptom severity compared to men. Some studies, however, reported a lack of or only a small association of sex on HRQoL (5, 69–72). Others support our findings that women report lower HRQoL compared to men (73–75), while others found higher HRQoL compared to women (76, 77). In the German general population, HRQoL was slightly lower in women compared to men (41). However, as the proportion of women was lower compared to men among alcohol use disorders (32.6% vs. 67.4%) and the proportion of mood affective disorders was higher (57.8% vs. 42.2%) in our study population, we conducted a sensitivity analysis including the diagnostic group in the regression analysis. The results showed that the lower HRQoL among women at measurement I was no longer significant (*p* = 0.311). All other variables remained in the same significance cluster (*p* ≤ 0.05 vs. *p* > 0.05), including sex at measurement II (data not shown).

Being not married or in co-habiting was associated with a decreased HRQoL during measurement I and with an increased HRQoL during measurement II in our study. An Ethiopian study revealed that being divorced was negatively associated with HRQoL among people with schizophrenia (78). However, due to cultural differences with the German population, these results are only comparable to a limited extent with our study population. Moreover, a study on severely mentally ill patients in Germany could not support those findings (5). One reason why being “not married” or in “co-habiting” was negatively associated during measurement I and positively during measurement II could be the possible different stages of the diseases at both time points. While measurement I was conducted during a clinical setting (inpatient, day care or outpatient) and therefore during a probably more acute phase of disease progression, measurement II was independent from acute treatment. However, further research is necessary in this area.

In our study, living alone was associated with lower HRQoL during measurement II, whereas living in supported accommodation did not reveal significant associations with HRQoL. Living alone and not being married or co-habiting are naturally highly correlated. Therefore, the findings during measurement II were congruent. However, the negative relationship during measurement I indicates that partnership status might add further explanation compared to considering living situation only. Another German study among patients with severely mentally ill patients found highest HRQoL among patients living in an assisted home (5). However, one reason for this difference could be that our study did not only include severely mentally ill patients. Less severely ill patients might be in lower need for supported living.

Lower education was associated with lower HRQoL in our study, which is in line with other studies (41, 79). Further, being incapacitated or unable to work was negatively associated with HRQoL during measurement II. This might reflect the relationship between the severity of the disease and occupational status. However, symptom severity was not associated with occupational status in our study. Other evidence also supports our finding that mentally ill persons with an occupation report better HRQoL (5, 80). Having one’s own income might be associated with financial security and autonomy, but also with having a social network (5, 80).

We found a negative association between HRQoL and chronic comorbidity, both at measurement I and measurement II. This result is supported by other research (41, 81, 82). Longer time in treatment was associated with lower HRQoL in our study during measurement I, whereas time in treatment was not associated with HRQoL during measurement II. In line with that finding, we found that longer time in treatment was also associated with higher symptom severity at measurement I.

### 4.6. Strengths and limitations

The PsychCare study is the first prospective, controlled, multi-perspective and multi-method evaluation study of FIT programs in Germany. It focusses on the perspectives of patients, which has not yet been considered in other studies evaluating FIT (83, 84). It thus adds important insight on the effects of such programs on patients. Another strength was the establishment of a control group, which was not used in other studies (85, 86). This study included patients from all settings in German psychiatric hospitals (inpatient, daycare and outpatient) and involved hospitals throughout Germany including hospitals with many years of FIT experience and those with less than 3 years of FIT experience by the time of patient recruitment onset.

Nonetheless, we also need to acknowledge some limitations. The COVID-19 pandemic, which led to significant inferences in mental health services, started during measurement II and probably distorted the results, as mentioned above. Further, some hospitals had difficulties to recruit study participants in all hospital settings. One reason for this was, as mentioned above, that the recruiting personnel was often only available for either inpatient, day care or outpatient treatment. This separation of the settings, especially in routine care, within German hospitals made it sometimes difficult to reach all patients, particularly those only experiencing outpatient treatment (15).

In addition, there were high rejection rates during the request for study participation among clinics in standard care (*n* = 14), which illustrates the difficulty to implement research in hospitals with TAU character (neither FIT nor university hospital). The general lack of personnel, especially in patient care, is a major limitation (87). FIT and university hospitals often have other personnel options (e.g., case managers) and structures (e.g., research coordination) supporting a successful recruitment. The number of patients included also fell short of expectations in some hospitals. Through harmonized training, follow-up training and close monitoring in the study, the targeted total number of cases at measurement I could, nevertheless, be achieved. In addition, the high loss-to-follow-up (63.5% and 74.4% vs. 25% estimated) is a limitation in this study. Furthermore, in various clinics, not all settings could be involved, particularly for some hospitals in the outpatient setting where recruitment was very low. This could influence the comparability between FIT and TAU. However, characteristics at measurement I and symptom severity hint to comparable groups in our analyses. Unfortunately, fewer patients than planned were reached during measurement II. One reason might be the severity of the illness at the time of study inclusion, which makes longitudinal research with (severely) mentally ill persons very difficult. Another reason might be that the recruitment process during measurement I was during hospital stay and measurement II was conducted *via* written questionnaires by the study centers. Patients might have higher motivation to participate being invited by the treating staff, which they know in person, instead of a non-related research team, which they have never seen.

Furthermore, measurement I was done in a clinical setting (inpatient, day care or outpatient), while measurement II was independent from the setting. We also did not control for phase of treatment (e.g., first treatment vs. longer duration of treatment). We expect the clinical pathways after study inclusion to be quite diverse. Due to the low number of patients participating in measurement II, outliers could distort our results.

In addition, we used self-reported data, which can lead to self-report bias, e.g., participants might feel obliges to provide socially desirable answers. However, on the one hand, this bias would occur in both groups (non-differential). On the other hand, we judge the outcomes HRQoL and symptom severity to be less prone to such a bias in contrast to, for example, satisfaction with care.

We purposely used a generic preference-based instrument to measure HRQoL. This allows us to compare the results across different diseases and use it for health-economic evaluations enriching evidence-based health policy decision-making. We are, however, aware that such generic instruments might not perfectly fit the targeted population. On the other hand, the QWB-SA has been found to be a valid instrument for our population under study (27, 53, 88). In addition, we could only include patients with sufficient command of the German language, as all study documents used were only in German. This might limit the generalizability of our study results to non-German speaking patients. However, we expect this limitation to be minor and present to the same extent in both FIT and TAU group.

The selection of potential co-variates were limited to the variables that we assessed in the context of the PsychCare study. Other factors such as self-esteem (5), self-stigma (89), self-efficacy (90), illness insight (91), or pharmaceutic side effects (91) etc., which are important factors influencing HRQoL could not be considered. Some severely ill patients could not be included; therefore, the HRQoL and symptom severity is likely to be underestimated. However, this was true for both the FIT and TAU group and should not infer with the group comparison.

## 5. Conclusion

In sum, HRQoL in our study was, at the time of recruitment during hospital treatment, higher among patients treated in FIT hospitals compared to patients in routine care, while symptom severity was comparable between both groups. HRQoL increased and symptom severity decreased from the time of recruitment to 15 months later. However, the difference between FIT and TAU observable during time of recruitment diminished 15 months later. Symptom severity remained comparable between both groups 15 months after recruitment. The dimension *acute and chronic symptoms* was associated with the highest detriments in HRQoL in both groups. We identified risk/protective factors associated with lower quality of life and higher symptom severity in both groups. We confirmed that HRQoL was negatively associated with symptom severity.

While HRQoL alone should not define the effect of an intervention, HRQoL is an important patient-centered outcome alongside with other outcomes, such as treatment satisfaction or recovery. They are necessary outcomes for a patient-centered evaluation of an intervention effect, such as the introduction of FIT with a GBT seen in Germany. The question asked in German politics is whether FIT hospitals provide better results to overcome the fragmented system compared to routine care. If they do so, ways to integrate aspects of FIT into routine care are politically discussed. Effects visible in other studies on FIT, e.g., the shift from inpatient to day care or outpatient treatment, needs to be strengthened by patient-centered outcomes, such as HRQoL. This is the first study providing evidence of FIT treatment on the patient-centered outcome HRQoL compared to routine care and shows evidence whether to integrate FIT into routine care.

## Data availability statement

The original contributions presented in this study are included in the article/Supplementary material, further inquiries can be directed to the corresponding author.

## Ethics statement

The studies involving human participants were reviewed and approved by the Institutional Review Board (IRB00001473 and IORG0001076) of the Medical Faculty of the Technische Universität Dresden and at each site where a separate approval was mandatory. The patients/participants provided their written informed consent to participate in this study.

## Author contributions

AN drafted the manuscript. AN and BS coordinated the study. FB analyzed the data and created the figure. AN, BS, RK, IW, JS, and AP contributed to the study design. AP and JS were principle investigators. AN, BS, RK, IW, JS, and FB contributed to the data interpretation. All authors read and commented on the manuscript and approved the final version of the manuscript.
