# Supplementary material for: Health-related quality of life among patients with treated alcohol use disorders, schizophreniform disorders or affective disorders and the influence of flexible and integrative psychiatric care models in Germany (PsychCare)
Source: Front Psychiatry. 2023 Mar 31;14:1068087. doi: 10.3389/fpsyt.2023.1068087 (PMC10102371; doi:10.3389/fpsyt.2023.1068087)
Supplement: Supplementary file 1 [file Table_1.DOCX]

# Supplementary Material

Table A: Health utility weights at measurement I, by setting

|  | **inpatient (N=574)** | **day care (N=413)** | **outpatient (N=141)** |
| --- | --- | --- | --- |
| **Health utility weights** |  |  |  |
| Mean (SD) | 0.484 (0.155) | 0.522 (0.146) | 0.558 (0.146) |
| Median [Min, Max] | 0.481 [0.0930, 1.00] | 0.533 [0.0930, 0.982] | 0.592 [0.159, 0.970] |

Table B: Health utility weights and symptom severity scores at measurement I and measurement II, by diagnosis at study entry, for participants who participated in measurement I and II

|  | **Measurement I**  Only participants who participated in measurement I and II | |  |
| --- | --- | --- | --- |
|  | **FIT** | **TAU** | **p-value** |
| **Mean health utility weights (SD)** | **n=209** | **n=135** |  |
| **Overall** | 0.538 (0.155) | 0.487 (0.156) | 0.003 |
| **by diagnosis at study entry** |  |  |  |
| alcohol use disorders | 0.593 (0.155) | 0.532 (0.128) |  |
| schizophreniform disorders | 0.539 (0.165) | 0.509 (0.174) |  |
| affective disorders | 0.518 (0.148) | 0.468 (0.158) |  |
| **Mean symptom severity (SD)** | **n=202** | **n=133** |  |
| **Overall** | 21.0 (8.03) | 22.0 (7.49) | 0.240 |
| **by diagnosis at study entry** |  |  |  |
| alcohol use disorders | 18.2 (7.36) | 17.7 (5.35) |  |
| schizophreniform disorders | 17.9 (7.41) | 18.1 (5.92) |  |
| affective disorders | 23.1 (7.84) | 24.3 (7.45) |  |

FIT = participants from FIT hospitals (flexible and integrated treatment), TAU = participants from routine care; diagnosis at study entry (ICD-10): alcohol use disorders (ICD-10: F10) = mental and behavioral disorder due to use of alcohol, schizophreniform disorders (ICD-10: F20-23) = schizophrenia, schizotypal disorder, delusional disorder or brief psychotic disorder
